# Relationship Between Vitamins and Diabetes

**DOI:** 10.7759/cureus.36815

**Published:** 2023-03-28

**Authors:** Devanshu S Raghuvanshi, Swarupa Chakole, Mayank Kumar

**Affiliations:** 1 Community Medicine, Jawaharlal Nehru Medical College, Datta Meghe Institute of Higher Education and Research, Wardha, IND; 2 Pharmacology, Jawaharlal Nehru Medical College, Datta Meghe Institute of Higher Education and Research, Wardha, IND

**Keywords:** antioxidant, insulin sensitivity, immunity, vitamins, diabetes

## Abstract

This review article aims to examine the relationship between specific vitamins and type 2 diabetes. Individuals with diabetes have been observed to have lower levels of specific antioxidant vitamins such as A, C, and E, possibly due to the need to manage oxidative stress caused by glucose metabolic abnormalities. Retinol-binding protein, which has adipocytokine activities, has a modulatory effect. Levels of thiamine, pyridoxine, and biotin are also lower in individuals with diabetes. While the reasons for this are unclear, some improvement in metabolic control has been observed with supplementation. Although metformin is the preferred treatment for type 2 diabetes, it has been found to limit the absorption of certain nutrients, including vitamin B9 and vitamin B12, necessitating regular supplementation of these nutrients for people with diabetes. Diabetes and its consequences, including cardiovascular disease, are more likely in those with low vitamin D levels. Although some research suggests that vitamin K intake may improve glucose metabolism, further evidence is required. Research on the effectiveness of multivitamins has produced inconsistent results, and there are no clear guidelines for vitamin supplementation in individuals with type 2 diabetes mellitus. However, people who use metformin for extended periods may benefit from additional folic acid and vitamin B12 supplements.

## Introduction and background

The global industry for dietary supplements is booming. Over $40 billion [1] and over $110 billion [2] have been predicted as total revenues for all applications of these items in the United States and the world, respectively. Despite the lack of proof for many commercially available supplements, people still like to use them. People may take supplements to improve their health, make up for a nutritional deficiency, or treat a specific health condition. Dietary supplements are regulated in the United States in a way that is very different from how drugs are regulated [3]. The authorities require manufacturers to prove the efficacy and safety of a drug before it can be on the market. The FDA will review the evidence and decide if the drug can be sold. Dietary supplements do not have to meet the same standard. By law, dietary supplements cannot be used to diagnose, treat, stop, or cure any disease. So, supplements can have their safety and effectiveness proven by the FDA before they can be sold. In addition, there are no rules concerning the authenticity of product claims. The sole restriction is that health supplement advertising cannot make any health claim whatsoever [4].

A supplement manufacturer, for instance, is not permitted to advertise their product as a diabetic cure. However, they can state that the product maintains normal blood sugar levels without implying that it treats high blood sugar. In real life, it can be challenging for people to tell the difference between these things so they can understand what might happen if they take a supplement [5]. New medications to treat diabetes mellitus (DM) keep appearing in response to the disease's prevalence in the global healthcare system. The increasing prevalence of type 2 diabetes, however, makes it critical to discover effective new preventative measures. Vitamin D has become more popular as a possible way to lower the risk of getting diabetes. According to the findings of plenty of recent studies, vitamin D may be responsible for crucial benefits outside of bone health. Not only are vitamin D receptors present in skeletal cells, but they are also present in beta cells, which may be found in the pancreas. Several distinct types of cells can potentially contain the vitamin D receptor. Vitamin D has been proven to lower the chance of developing type 2 diabetes, and more recent studies confirm this assertion [5]. Some of the causes of this are the effects of vitamin D on beta cell activity, insulin sensitivity, and systemic inflammation. Most information comes from studies examining how vitamin D levels affect the progression of type 2 diabetes or how they affect glycemia in people who already have diabetes. Newer, smaller, randomized trials on the effects of vitamin D supplementation on diabetes risk and blood sugar levels have shown mixed results, whether the supplement is taken with or without calcium [3].

## Review

Methods

**Figure 1:**
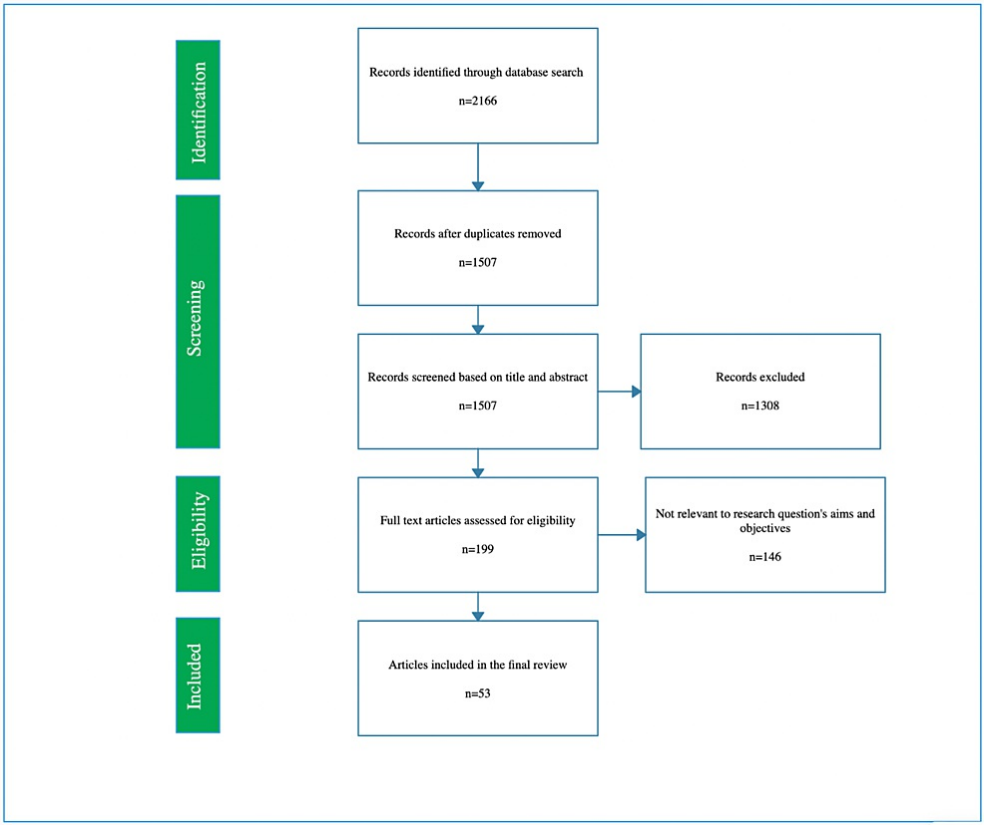
PRISMA flowchart for search. PRISMA: Preferred Reporting Items for Systematic Reviews and Meta-Analyses. Image credit: Authors.

The terms ‘vitamins’, ‘diabetes’, ‘insulin sensitivity’, ‘immunity’, ‘antioxidants’, and ‘type 2 diabetes’ were searched for in a database like ‘PubMed’. Only results pertaining to the English language were shown. If there was more than one published report from a similar study, the latest one was used. Only review articles that also had original data were taken into account. The PRISMA (Preferred Reporting Items for Systematic Reviews and Meta-Analyses) for search is shown in Figure 1.

Vitamin A

The term "vitamin A" describes a class of compounds with structural and functional similarities. Retinol is the most active form of vitamin A and is esterified with long-chain fatty acids in animal tissues. Enzymes break down carotenes found in plant cells into retinal, which is then changed into retinol in the enterocyte. Retinol can also be made from some xanthines. Vitamin A is involved in many metabolic processes, such as the expression of genes, the separation of cells, and their growth. It is vital to the body's defense mechanisms, fetal growth, senses of sight, smell, taste, hearing, hunger, and sperm production. Because of their antioxidant properties, retinoids play a crucial role in maintaining homeostasis in a stressed body [6]. Lipid metabolism in the liver, adipogenesis, and the functioning of pancreatic cells have all been speculated to have a role for retinoids. Insulin sensitivity is significantly impacted by the adipokine retinol-binding protein (RBP), which transports retinoids. Improved lipid profiles are shown in a mouse model lacking the gene for retinaldehyde dehydrogenase 1 (Raldh1a1), which is necessary to produce retinoic acid for lipid metabolism [7].

It is clear that an average healthy person, and especially those with long-term diseases involving carbohydrates and lipids, should keep up their vitamin A intake and concentrations. However, more investigation is required to understand how retinoids and their mechanisms affect carbohydrate and lipid metabolism in well-being and disease. Those with advanced age and type 2 diabetes have reduced amounts of vitamin A and carotenoids in their blood [8]. Diabetic adults, on the other hand, have normal retinol levels in their blood but have lower carotene and RBP levels than controls [9]. After accounting for other risk factors for cardiovascular disease, structured case-control research found that those with higher blood levels of beta-carotene had a decreased chance of developing diabetes [10]. Researchers have also found that diabetic people with nephropathy have more retinol in their urine than people who do not have diabetes [11]. Diabetes problems such as retinopathy, cardiovascular problems, nephropathy, and non-alcoholic fatty liver have been associated with RBP4 in several research. However, the results are still questionable.

While it is true that the ratio of RBP concentrations to retinol blood concentrations is high in individuals with diabetes, the explanation for this is yet unknown. When comparing patients with type 2 diabetes, adequate or impaired glucose tolerance, and obesity, Erikstrup et al. [12] found that those with diabetes had relatively low levels of RBP and retinol and a higher RBP-to-retinol ratio. The retinol/RBP ratio and retinol levels have both been shown to be higher in diabetes patients than in controls, suggesting an overabundance of retinol in this population [13]. Improved insulin sensitivity has been shown in diabetic mice after treatment with retinoic acid [14], which decreases the ratio of retinol to retinol-binding protein 4 (RBP4).

Vitamin B

Type 2 diabetes mellitus has been linked to many B vitamins, including vitamins B1, B2, B3, B5, B6, B7, B12, and B9. However, the evidence is weakest for B2 and B5. Thiamine (B1) serves as a coenzyme that performs various functions, which include aiding in the exchange of aldehyde groups and glycation. Additionally, it plays a role in transmitting signals in the nervous system and the conduction of nerve impulses. These actions are of significant importance and have the potential to influence the onset of diabetes, as stated in reference [15]. According to a study conducted by Polizzi et al. [16], individuals with diabetes and nephropathy exhibited elevated levels of DNA glycation in their leukocytes. However, after undergoing a five-month treatment involving thiamine and pyridoxine supplements, the levels of DNA glycation decreased. Both type 1 and type 2 diabetes have been reported to have low thiamine levels and higher renal clearance [17]. In a cross-sectional investigation, thiamine levels were shown to be lower in people with diabetes compared to healthy controls, DM patients with microalbuminuria, and DM patients with macroalbuminuria. Researchers have discovered an inverse relationship between thiamine levels and lipid profile [18] in microalbuminuria.

Vitamin B6 is made up of three different molecules: pyridoxal, pyridoxine, and pyridoxamine, along with their phosphorylated derivatives. In its accessible form, pyridoxal-5'-phosphate, vitamin B6 is essential for normal bodily function (pyridoxal 5'-phosphate (PLP)). Like an aminotransferase and as a cofactor for glucose phosphorylase, it plays a crucial role in the metabolism of glucose [19], which is necessary for the utilization of glycogen in the liver and muscles. Diabetic individuals with a recent diagnosis had lower PLP concentrations than healthy controls [20]. Although a long-term placebo-controlled study of combined folate, pyridoxine, and B12 medication did not reveal significant improvements in the likelihood of acquiring type 2 diabetes in women who were at high risk of cardiovascular events, it did find a drop in homocysteine levels [21].

Niacin supplementation improves high-density lipoprotein (HDL) cholesterol while lowering triacylglycerides and low-density lipoprotein (LDL) cholesterol [22,23], although the impact of diabetes on these parameters has received less attention. It is a lipid-lowering medication used alone or in conjunction with others, although its effectiveness in reducing the risk of cardiovascular disease is debatable [24]. The creation of methionine, pyrimidine, and purine bases all require vitamin B12, which functions as a coenzyme in the metabolic pathways that include single-carbon molecules. Its deficiency, which can result in DNA damage or improper repair, has been linked to cancer, vascular disorders, and certain birth abnormalities. Hyperhomocysteinemia, which is associated with folic acid deficiency, is also a known contributor to hypertension and atherosclerosis [25]. Due to its widespread availability in animal products, vitamin B12 insufficiency is unusual in the general population but more prevalent among vegetarians. However, the risk of cobalamin deficiency is raised by the long-term use of metformin, the treatment of choice in uncomplicated diabetes [26-31]. Type 2 diabetes on metformin has been linked to reduced levels of vitamin B12 in the blood, according to cross-sectional research and a retrospective assessment of their medical records [32,33]. Metformin medication, even when administered for a very short period, has been shown to reduce cobalamin levels in the elderly [34]. In contrast, vitamin B12 insufficiency has been recorded in people with diabetes who are not on metformin [35]. Metformin-treated diabetes fared cognitively worse than their non-treated or metformin-naive counterparts. The authors recommend using vitamin B12 to boost mental capacity [36]. Vitamin B12 metabolic indicators were examined both inside and outside of cells by Obeid et al. Vitamin B12 levels were shown to be normal extracellularly but low intracellularly in those with type 2 diabetes; however, metformin treatment restored this [37].

A deficiency of folic acid, which can be found in animal products, leafy greens, legumes, and nuts [38], has been associated with several health conditions, including megaloblastic anemia, neural tube defects, cardiovascular disease, cancer, and senile dementia. Although vitamin B12 deficiency and the resulting hyperhomocysteinemia are not common, supplementing experiments have been conducted in people with diabetes because of the role folic acid plays in the etiology of the disease. Hyperhomocysteinemia has been linked to inadequate folate and B-12 intakes in individuals with type 2 diabetes, according to a case-control study [36]. Folic acid reverses DNA damage, which can be measured by the appearance of micronuclei. Individuals with diabetes experience reduced impact from oxidative stress due to this phenomenon [39]. Studies have shown that taking additional folate can help people with type 2 diabetes better control their blood sugar levels by lowering glycosylated hemoglobin, fasting glucose, insulin levels, insulin resistance, and homocysteinemia [40]. This is accomplished by lowering the amount of folate the body converts into homocysteine.

Vitamin C and E

Studies have revealed that individuals with diabetes have lower levels of antioxidants, vitamin C, and vitamin E than healthy individuals [41]. Furthermore, research has indicated that lipid peroxidation levels increase while antioxidant enzymes, vitamin C, and vitamin E levels decrease within the first two years of a type 2 DM diagnosis [42]. Interestingly, plasma vitamin C levels are inversely related to glycated hemoglobin, fasting and postprandial blood glucose, and peroxidation levels [43,44]. However, lipid profiles appear to be unrelated to these factors. These findings suggest that adequate levels of antioxidants and vitamins C and E may be essential for managing diabetes and its complications.

Individuals who have a clinical diagnosis of type-2 diabetes and periodontitis have demonstrated positive results in the management of their chronic periodontitis symptoms following a combination of vitamin C and dental operations [45]. Poor dental health serves as a risk factor for diabetes and also indicates the presence of diabetes-related periodontal disease. Furthermore, vitamin C has been found to alleviate anxiety in people with diabetes, but it does not have an effect on stress or depression [46]. Studies have shown that taking vitamin C and E supplements for three months resulted in lowered blood pressure and glucose levels and an increase in superoxide dismutase and glutathione [47].

Vitamin D

Studies on humans have brought up the possibility of a connection between vitamin D and either form of diabetes, with the evidence supporting the connection between vitamin D and type 2 diabetes being greater. However, it is essential to keep in mind that nearly all of the data that are currently accessible on type 2 diabetes in humans come from randomized clinical trials, which are susceptible to flaws in design and methodology and are unable to establish a causal relationship [48]. For this reason, it is essential to keep that data in mind. Vitamin deficiency has been linked to insulin resistance, which is a key factor in the development of type 2 diabetes. Insulin resistance means that the body's cells are less responsive to the insulin produced, increasing blood glucose levels. In both animal and human studies, vitamin D has been shown to improve insulin sensitivity and glucose metabolism [48].

Moreover, a deficiency of this vitamin is also associated with an increased risk of developing diabetes complications, such as coronary heart disease, retinopathy, and neuropathy. Therefore, maintaining adequate vitamin D levels through sunlight exposure, dietary intake, and supplementation may play a role in preventing and managing type 2 diabetes and its complications. However, further research is needed to determine the optimal vitamin D levels and the most effective ways to supplement vitamin D in people with diabetes [49,50]. These failures have resulted in the adoption of new therapeutic approaches. Therefore, data from randomized controlled trials are required to completely analyze the preventative benefits of this vitamin on type 2 diabetes and to resolve the question of causation [49].

Vitamin K

Vitamin K, composed of phylloquinone and menaquinone, is found in a wide variety of animal and plant products [50]. According to some studies [51], vitamin K consumption is associated with improved insulin sensitivity, glucose metabolism, and a lower likelihood of developing diabetes. One study in Spain investigated whether or not there was a correlation between the use of vitamin K and the manifestations of diabetes [51]. Initially, the researchers discovered no link between the two. However, after tracking the individuals for a year, those with the greatest vitamin K intakes were shown to have lower levels of hormones associated with hunger and fat storage and a decreased chance of developing diabetes mellitus [51,52]. Another retrospective research was conducted in the Netherlands and found that intakes of phylloquinone and menaquinone were inversely related to the risk of acquiring type 2 diabetes mellitus [53].

## Conclusions

Even though vitamins have a big effect on the risk of DM, the way it develops, and the effects of the disease, there is not always enough evidence to recommend that people with diabetes take them on their own or in groups. It is suggested that enough vitamin-rich foods be eaten to keep a person's nutritional status up to par. To identify specific intake deficiencies and provide appropriate recommendations, it is necessary to conduct nutritional evaluations. Taking into account how the supplement affects the diet as a whole brings up the risk of too much of a certain vitamin or toxicity. There is sufficient scientific evidence to recommend the use of metformin in individuals with type 2 diabetes, even though vitamin B12 supplementation is not required for metformin to reduce the incidence of neuropathy and its associated consequences.
